# Lack of reproducibility of linkage results in serially measured blood pressure data

**DOI:** 10.1186/1471-2156-4-S1-S37

**Published:** 2003-12-31

**Authors:** Sanjay R Patel, Juan C Celedon, Scott T Weiss, Lyle J Palmer

**Affiliations:** 1 Channing Laboratory, Brigham and Women's Hospital and Harvard Medical School, Boston, Massachusettes, USA; 2 Western Australian Institute for Medical Research and Centre for Medical Research, University of Western Australia, Perth, Australia

## Abstract

**Background:**

Using the longitudinal Framingham Heart Study data on blood pressure, we analyzed the reproducibility of linkage measures from serial cross-sectional surveys of a defined population by performing genome-wide model-free linkage analyses to systolic blood pressure (SBP) and history of hypertension (HTN) measured at five separate time points.

**Results:**

The heritability of SBP was relatively stable over time, ranging from 11.6 to 23.5% (coefficient of variation = 25.7%). However, the variability in linkage results was much greater. The average correlation in LOD scores at any pair of time points was 0.46 for HTN (NPL All LOD) and 0.17 for SBP (Variance Components LOD). No evidence of reproducible linkage results was found, with a mean κ of 0.02 for linkage to HTN and -0.03 for SBP linkage. At loci with potential evidence for linkage (LOD > 1.0 at one or more time points), the correlation was even lower. The coefficient of variation at loci with potential evidence of linkage was 126% for HTN and 135% for SBP. None of 15 chromosomal regions for HTN and only one of 28 regions for SBP with potential evidence for linkage had a LOD > 1.0 at more than two of the five time points.

**Conclusion:**

These data suggest that, although heritability estimates at different time points are relatively robust, the reproducibility of linkage results in serial cross-sectional samples of a geographically defined population at successive time points is poor. This may explain in part the difficulty encountered in replicating linkage studies of complex phenotypes.

## Background

The inability to replicate linkage findings has been a significant limitation in the advancement of understanding the genetics of complex human diseases [1]. This problem in reproducibility has been attributed to high false-positive rates due to the large number of simultaneous hypotheses being tested in genome screens, to differences in study design and phenotype definition, genetic heterogeneity, or to differences in environmental exposures affecting gene × environment interactions [2]. In addition, within a defined population, the strength of linkage to genes that regulate the rate at which a phenotype changes over time (i.e., natural history) or that have age-dependent effects may depend on the point in time at which phenotypic measurements are made because of changes in the distribution of ontogenetic stages or ages.

Although the study of complex disease genetics has thus far almost exclusively relied on the use of phenotypic data obtained at a single time point by a cross-sectional study, it is reasonable to hypothesize that longitudinally collected data may provide more insight into genetic susceptibilities for disease. Methodologies for the analysis of phenotypic data collected at multiple time points remain under development. An understanding of the reproducibility of linkage results using phenotypic data measured at different time points in serial cross-sectional surveys of a defined population will be important both to our understanding of the potential importance of sampling variation between studies and in developing robust methods for analyzing longitudinal data. The longitudinal Framingham Heart Study data made available through GAW13 allows these questions to be directly addressed. In this paper, we have explored the stability of linkage results to both a dichotomous and a quantitative trait in serial cross-sectional samples of a population-based sample in order to better understand the dependence of analytic results on sampling error and the particular time at which a sample is phenotypically assessed.

## Methods

The original (parent) cohort had phenotypic data regarding blood pressure collected at 21 time points spaced 2 years apart over 40 years while the offspring cohort was studied over 20 years at five time points every 4 years apart except for 8 years between the first two time points. To generate populations with maximal power, the parent and offspring cohorts were combined for this analysis. For each time point at which there were offspring data, phenotypic data from the closest date in the parent data set were used. This resulted in combining the 1971, 1979, 1983, 1987, and 1991 offspring data with the 1972, 1978, 1982, 1986, and 1988 parent data. The traits of interest were a diagnosis of hypertension (HTN) and systolic blood pressure (SBP). HTN was defined as systolic blood pressure ≥ 140 mm Hg, diastolic blood pressure ≥ 90 mm Hg, or use of medical therapy for hypertension. For those who were receiving antihypertensive medications, the SBP was assumed to be 10 mm Hg greater than the measured SBP as data suggest that this is the average reduction seen with medical therapy [3,4]. This "correction factor" for SBP has also been found to usefully recover the genetic information in subjects receiving antihypertensive treatment [5,6].

We used the Whittemore and Halpern NPL (nonparametric linkage) all statistic to test for allele sharing among all hypertensive individuals in a pedigree [7]. The Kong and Cox linear model was used to calculate a nonparametric LOD score [8]. This process was repeated for each of the five time points of data collection.

To test for allele sharing using SBP as the quantitative trait of interest, a variance components model was constructed for each of the five time points. The total phenotypic variance (conditional on the mean model) was based on a conventional covariate structure appropriate to the extended families present in the Framingham cohort. The model specified was:

σ^2^_Total _= σ^2^_A _+ σ^2^_CS _+ σ^2^_C _+ σ^2^_E_.

In this model, σ^2^_A _represents additive genetic effects, σ^2^_CS _the effects of common sibling environment, σ^2^_C _the effects of a common family environment, and σ^2^_E _the residual variance (which is assumed to arise from nonfamilial factors). The narrow sense heritability (h^2^_N_) was calculated as σ^2^_A _/ σ^2^_Total_. Age, gender, alcohol use (g/day), smoking (cigarettes/day), height, weight, and fasting glucose were included in the model as possible fixed effects. Linkage to the locus of interest was tested by comparing the likelihood of a model where the variance due to the locus of interest was constrained to zero versus an unrestricted model.

The linkage analyses of HTN were undertaken using the program MERLIN [9]; variance components analyses of SBP were undertaken in SOLAR [10]. Whole-genome, multi-point linkage analyses at 1-cM intervals of HTN and SBP were performed using 394 polymorphic markers on the 22 autosomes for each time point. Potential evidence of linkage was defined as LOD > 1.0 at ≥ 1 time point. Although this threshold does not meet Lander and Kruglyak's criteria for genome-wide significance [11], LOD > 1 is commonly used as a measure of promising evidence for linkage in the context of a complex disease [12].

The coefficient of variation (CV) of the heritability over the five time points was calculated to assess the reproducibility of this measure of additive genetic effect. To assess the reproducibility of linkage findings, Spearman correlations of LOD scores between each pair of time points were calculated over the entire genome as well as over the subset of loci where evidence for potential linkage was observed. In addition, the CV was calculated at each locus over the five time points to describe the variability in the LOD scores for linkage with each of the two traits. Mean and SD of the CVs at those loci where evidence for potential linkage was found are reported. The evidence for linkage was dichotomized at a threshold of LOD = 1.0 and Cohen's kappa statistic [13] was calculated between each pair of time points to provide another estimate of the reproducibility of results.

## Results

The characteristics of the population are shown in Table 1. The sample size decreased over time, with the decline from the first to the last time point amounting to a 27% reduction. Concomitantly, the variability in SBP rose, as did the prevalence of HTN. The mean Pearson correlation for SBP between each pair of time points was 0.69 and the mean κ for HTN status was 0.55.

As is shown in Table 2, the calculated heritability of SBP was relatively stable over time, ranging from 11.6% to 23.5% with no clear trend over time. The CV for SBP heritability was 25.7%.

**Figure 1:**
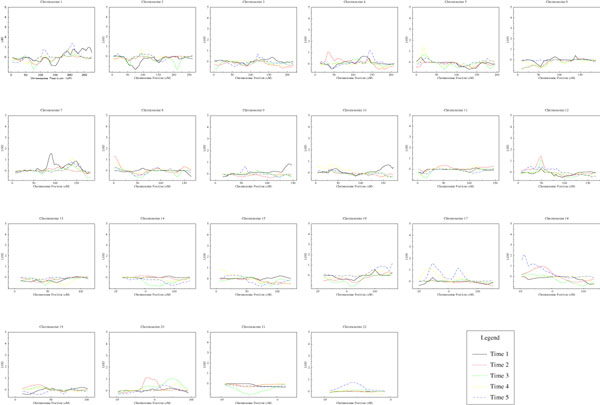
NPL-all linkage analyses (MERLIN) of hypertension at five time points.

The results of the NPL analysis of HTN for the five time points are displayed in Figure 1. The correlations in LOD scores between each pair of time points are shown in Table 3. Overall, the correlation was low to moderate, with an average correlation of 0.46. As expected, the correlation in linkage evidence decreased with increasing distance between two time points. The correlation was only 0.03 in the subset of loci where potential evidence of linkage existed. Using a dichotomous definition of linkage (LOD ≥ 1 versus LOD < 1), κ statistics for reproducibility of linkage evidence were also calculated (Table 4). The average κ was only 0.02, again suggesting poor reproducibility of linkage. A total of 15 chromosomal regions displayed evidence of potential linkage (LOD ≥ 1), with nearly half of these peaks occurring at the last time point. Only two of these 15 regions (12p and 17p) had a peak LOD > 1 at two different time points and no region had peak LOD > 1 at three or more of the five times. The average CV in LOD score over these 15 regions was 126%.

**Figure 2:**
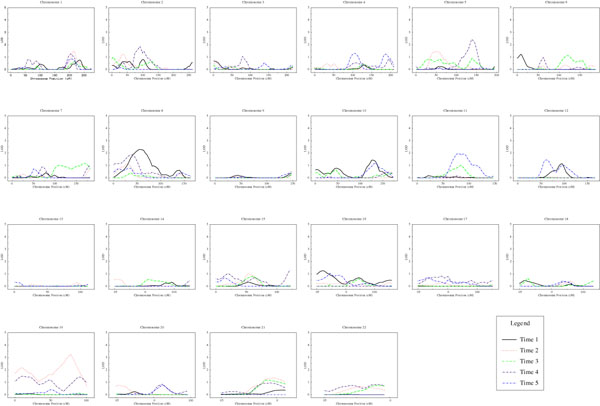
**Variance components linkage analyses (SOLAR) of SBP at five time points. **Models adjusted for age, sex, height, weight, smoking, and alcohol consumption.

The results of the variance components linkage analysis for SBP are plotted in Figure 2. The correlation matrix comparing the five time points is given in Table 5. It is clear that the correlations are poor to nonexistent with a mean correlation of only 0.17. Among the loci with evidence of potential linkage, the mean correlation was -0.03. Similarly, the κ statistics displayed in Table 6 demonstrates poor reproducibility of linkage, with a mean value of only 0.05. A total of 28 chromosomal regions had LOD > 1 for SBP at one time point. Nine of these 28 regions had evidence of potential linkage at two different time points and only one region (8p) had a peak LOD > 1 at three of the five times. The mean CV in LOD score over these 28 regions was 135%.

## Discussion

Our findings suggest that although heritability measurements are relatively stable over time, the finding of linkage to either a quantitative or dichotomous trait using cross-sectional data is highly dependent on the particular time point at which phenotypic data is collected. Evidence for linkage was markedly more labile when potential linkage (LOD > 1.0) was present on at least one time point. This may in part be due to regression to the mean but the poor reproducibility overall suggests other mechanisms are in play.

Part of the explanation for the high degree of variability in linkage evidence may be a loss of power as the number of phenotyped individuals fell with time. However, the loss was not dramatic and in addition, over half of the instances of LOD > 1.0 for both traits occurred in the last two time points. Alternatively, the lack of reproducibility may reflect the importance of genes whose effects are highly dependent on age or on gene × environment interactions. The disparate results of the linkage analyses at time points 3 and 4, where the average age is only 2 years apart, the sample sizes are within 3%, and the distribution of SBP and HTN are similar, counter this argument.

Although there is variability in individual SBP and HTN assessments in this cohort, the correlations in these phenotype measures was much stronger than that found in linkage findings. Importantly, even analyses of highly variable phenotypic measures over time would not necessarily imply poor reproducibility in linkage results so long as the familial correlations in the measures remain relatively constant (as was suggested by the relative stability of the heritability estimates for SBP over time).

The changing composition of the population over the five time points may have also contributed to the variation in linkage findings. One would expect the correlation to be greater if the identical population was studied at each time point. We deliberately did not restrict our analysis to pedigrees for which there were data at all time points both because this was not the primary hypothesis we wished to test and because we would have substantially limited our power to detect linkage. We also believe our approach more accurately reflects the data that would actually be available to researchers studying longitudinal populations. Assuming that the population studied at each time point is a random sampling of the general population of the town of Framingham, the linkage results for each cross-sectional study should represent an unbiased estimate of the true evidence for linkage in the entire population.

A high degree of lability in complex phenotypic measures over time together with simple sampling error may be the most likely explanations for our findings. Stochastic processes may have a larger influence on complex phenotypes, and hence on the results of linkage studies, than has been previously appreciated. The implications of this are disturbing, given than most genetic linkage and association studies have been based on cross-sectional surveys. It is possible that phenotypic variability and random error may be restricted in highly ascertained samples, reducing this problem. However, in the absence of empirical evidence of this, given the high caliber of the Framingham Heart Study in terms of both design and quality of phenotyping, it is difficult to argue that other studies would be less subject to this phenomenon. Our results highlight the difficulty of replicating linkage results for complex phenotypes and make the need for large longitudinal studies of such phenotypes clear.

These findings may help to explain the difficulties that have been experienced in replicating linkage results from cross-sectional genome scans [1]. Our findings also suggest that further investigations of the lability of linkage evidence in other conditions are warranted, and highlight the need for developing robust methods for analyzing longitudinal data in the context of linkage [14].

## Figures and Tables

**Table 1:** Population characteristics

**Time Point**	**N**	**Mean Age (SE)**	**Mean SBP (SE) (mm Hg)**	**% HTN**
1	2563	43.5 (0.35)	127 (0.39)	27.6
2	2131	50.8 (0.35)	128 (0.44)	32.9
3	2027	53.5 (0.34)	130 (0.48)	39.1
4	1968	55.6 (0.34)	132 (0.55)	42.4
5	1868	58.5 (0.34)	132 (0.54)	39.9

**Table 2:** Heritability and linkage results over time

	**Time Point**				
	
	1	2	3	4	5
Heritability of SBP (%)	17.9	23.5	11.6	17.7	22.8
No. Regions with LOD > 1.0 for HTN	1	4	2	3	7
No. Regions with LOD > 1.0 for SBP	6	9	6	10	7

**Table 3:** Correlation of HTN LOD scores

**Time Point**	**1**	**2**	**3**	**4**	**5**
1	1	0.31	0.43	0.28	0.26
2		1	0.66	0.53	0.38
3			1	0.62	0.57
4				1	0.56
5					1

**Table 4:** Cohen's kappa for HTN LOD scores

**Time Point**	**1**	**2**	**3**	**4**	**5**
1	1	0.00	0.00	0.00	-0.01
2		1	0.10	-0.01	-0.01
3			1	-0.01	-0.01
4				1	0.11
5					1

**Table 5:** Correlation of SBP LOD scores

**Time Point**	**1**	**2**	**3**	**4**	**5**
1	1	0.10	0.21	0.01	0.17
2		1	0.25	0.41	0.14
3			1	0.27	-0.06
4				1	0.21
5					1

**Table 6:** Cohen's kappa for SBP LOD scores

**Time Point**	**1**	**2**	**3**	**4**	**5**
1	1	0.00	-0.02	0.11	0.08
2		1	0.10	0.34	-0.04
3			1	-0.02	0.00
4				1	-0.05
5					1
